# Age‐Dependent Clonal Expansion of Non–Sperm‐Forming Spermatogonial Stem Cells in Mouse Testes

**DOI:** 10.1111/acel.70019

**Published:** 2025-02-22

**Authors:** Terumichi Kawahara, Shinnosuke Suzuki, Toshinori Nakagawa, Yuki Kamo, Miki Kanouchi, Miyako Fujita, Maki Hattori, Atsuko Suzuki, Kentaro Tanemura, Shosei Yoshida, Kenshiro Hara

**Affiliations:** ^1^ Graduate School of Agricultural Science Tohoku University Sendai Japan; ^2^ Division of Germ Cell Biology, National Institute for Basic Biology National Institutes of Natural Sciences Okazaki Japan; ^3^ Graduate Institute for Advanced Studies, SOKENDAI Okazaki Japan; ^4^ Advanced Research Division for New Fields Within a Higher Research Organization Tohoku University Sendai Japan

**Keywords:** clonal expansion, lineage tracing, open niche, spermatogenic failure, spermatogonial stem cells, stem cell aging, stem cell heterogeneity, stem cell migration

## Abstract

In male mammals, spermatogonial stem cells (SSCs) are essential for sustaining lifelong spermatogenesis within the testicular open niche, a unique environment that allows SSC migration over an extended niche area. As SSCs undergo continuous mitotic division, mutations accumulate and are transmitted to the descendant SSC clones. Therefore, SSC clonal fate behaviors, in terms of their efficiencies in completing spermatogenesis and undergoing expansion within the niche, influence sperm genomic diversity. We aimed to elucidate the effects of physiological aging on SSC clonal fate behavior within the testicular open niche. We used single‐cell RNA sequencing, lineage tracing, and intravital live imaging to investigate SSC behavior in aged mouse testes, where spermatogenesis, although reduced, persists. We found that undifferentiated spermatogonia maintained gene expression heterogeneity during aging. Among these, GFRα1^+^ cells, which exhibited state heterogeneity, showed accelerated proliferation and persistent motility, continuing to function as SSCs in older mice. In contrast, a subset of SSCs characterized by low *Egr4* and *Cops5* expression did not contribute to spermatid formation. These non–sperm‐forming SSC clones increased in proportion among the total SSC clones and expanded spatially within the testicular open niche in old mice, a phenomenon not observed in young mice. The expansion of non–sperm‐forming SSC clones in aged testes suggests that they occupy a niche space, limiting the availability of functional SSCs and potentially reducing sperm production and genetic diversity. These findings highlight age‐specific clonal characteristics as hallmarks of stem cell aging within the testicular open niche and provide novel insights into the mechanisms governing reproductive aging.

## Graphical abstract

We found that GFRα1^+^ spermatogonial stem cells (SSCs) with reduced Egr4 and Cops5 expression did not form spermatids in mouse testes. Furthermore, the proportion of their clones relative to the total SSC clones increased and their distribution area expanded in old mice.

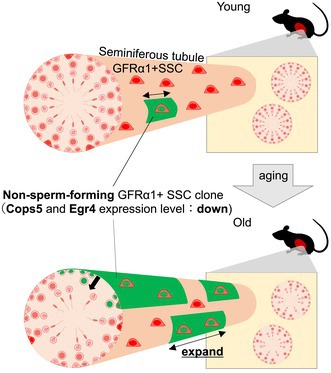

## 1 Introduction

In male mammals, spermatogonial stem cells (SSCs) support spermatogenesis by balancing proliferation and differentiation while migrating along seminiferous tubules within the testes (de Rooij and Russell 2000; Yoshida 2018). As males age, repeated mitotic divisions of SSCs lead to the accumulation of mutations in their genomes, which are subsequently transmitted to clonal progenies within the testis. As the clonal fates of SSCs, in terms of their efficiencies in completing spermatogenesis and undergoing expansion within the niche, can either amplify or diminish the proportion of sperm carrying their genetic information elucidating SSC clonal fate behavior in aged testes is crucial to understanding their role in the productivity and genetic diversity of sperm in aged male mammals. However, little is currently known about the clonal fate behavior of SSCs in physiologically aged mouse testes.

In mice, males live for over 2 years (Yuan et al. 2012) and reach sexual maturity between 2 and 6 months of age (defined as “young age” in this study), during which steady‐state spermatogenesis begins. At approximately 1.5 years of age (“middle age”), no significant morphological changes in the seminiferous tubules are observed (Nakano et al. 2021). However, by 2 years of age (“old age”), sperm numbers decrease, although they are not completely depleted (Endo et al. 2024). This suggests that even in old mouse testes, SSCs continue to sustain spermatogenesis, albeit at a reduced level. Although the characteristics of SSCs in young testes are well established, studies on the characteristics of SSCs in physiologically aged mouse testes remain limited, posing a significant barrier to understanding how aging affects the clonal dynamics of SSCs in vivo.

**FIGURE 1:**
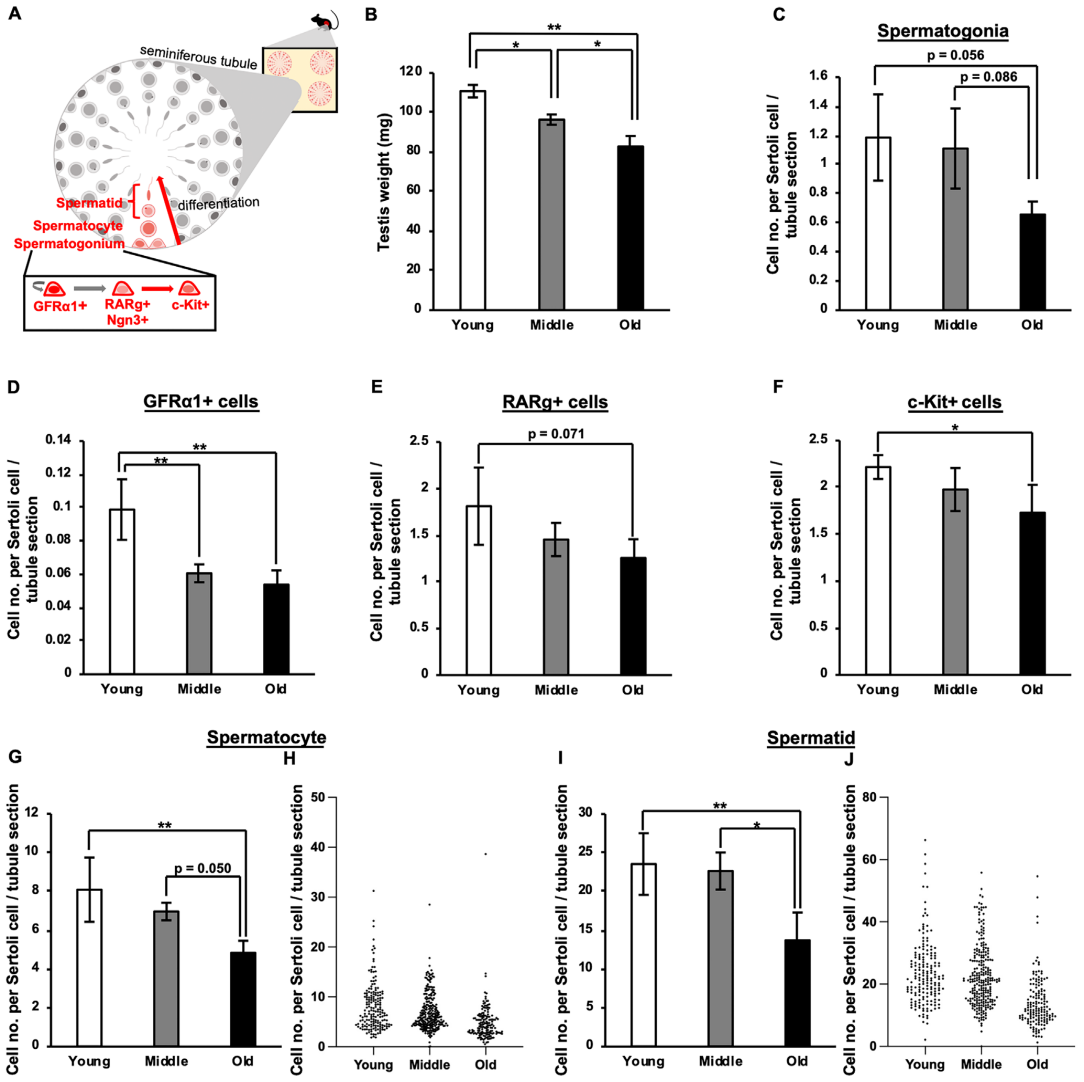
Age‐related changes in the number of testicular cells. (A) Schematic diagram of spermatogenesis illustrating the process from spermatogonia to spermatids within seminiferous tubules. In young mouse testes, GFRα1^+^ spermatogonia function as spermatogonial stem cells (SSCs), giving rise to Ngn3^+^/Rarg^+^ spermatogonia, which then differentiate into c‐Kit^+^ spermatogonia. (B) Age‐related changes in testis weight. Young, 3–4 months (16 testes from eight individuals); middle, 18–20 months (18 testes from nine individuals); and old, 25–28 months (14 testes from seven individuals). Values represent averages ± SD of individual data. *p < 0.05, **p < 0.01 (Tukey's multiple comparison test). (C) Age‐related changes in total spermatogonia density per seminiferous tubule cross section. (D–F) Age‐related changes in spermatogonia density at different differentiation stages per seminiferous tubule cross section: (D) GFRα1^+^, (E) Rarg^+^, and (F) c‐KIT^+^ cells. (G–J) Age‐related changes in the cell density of (G, H) spermatocytes and (I, J) spermatids per seminiferous tubule cross section. (H, J) Scatter plots showing the cell density per seminiferous tubule cross section from all individuals. (B–G, I) Values represent means ± SD. *p < 0.05, **p < 0.01 (Tukey's multiple comparison test). (C–J) Data exclude cross sections classified as Sertoli cell‐only (SCO) tubules. Cell density per seminiferous tubule cross section, defined as the number of cells per Sertoli cell, was analyzed for each individual (young [3 months]: 44, 44, 46, and 44 tubules from four individuals; middle [15–18 months]: 56, 60, 52, 45, and 49 tubules from five individuals; old [26 months]: 54, 53, and 47 tubules from three individuals). In (C–G, I), graphs show the mean cell density across individuals in each age group.

SSCs reside in a population of undifferentiated spermatogonia (A_undiff_). A_undiff_ includes singly isolated cells (A_single_ or A_s_) and the syncytia of two (A_paired_ or A_pr_) or more cells (A_aligned_ or A_al_) (de Rooij and Russell 2000). In young mice, within A_undiff_ subpopulations, the GFRα1‐expressing cells, comprising mainly A_s_ and A_pr_, with some A_al_, function as SSCs in young mice (Hara et al. 2014; Meng et al. 2000). In addition to morphological heterogeneity, GFRα1^+^ SSCs also exhibit heterogeneity in gene expression with different states primed for either self‐renewal (co‐expressing genes such as Plvap, Pdx1, Shisa6, or Eomes) or differentiation (co‐expressing genes such as Sox3, Rarg, or Ngn3) (Gely‐Pernot et al. 2012; La et al. 2018; Nakagawa et al. 2021; Raverot et al. 2005; Sharma et al. 2019a; Tokue et al. 2017; Yoshida et al. 2004). In contrast, the GFRα1^−^ subpopulation, expressing markers such as Rarg, Ngn3, and Piwil4 (Miwi2), consists mostly of A_al_ and fewer A_s_/A_pr_ and is primarily destined for differentiation into c‐Kit^+^‐differentiating spermatogonia (Carrieri et al. 2017; Gely‐Pernot et al. 2012; Ikami et al. 2015; Nakagawa et al. 2021, 2007, 2010; Yoshida et al. 2004). Upon differentiation, A_undiff_ gives rise to differentiating spermatogonia, followed by spermatocytes and eventually spermatids (de Rooij and Russell 2000; Russell et al. 1990) (Figure 1A). However, to date, state heterogeneity and stem cell function of GFRα1^+^ cells in the testes of old mice remain unevaluated.

The SSC niche, located on the basement membrane, represents a unique model of a stem cell niche as it lacks anatomical confinement and is referred to as an “open niche” (Stine and Matunis 2013; Yoshida 2018). Lineage‐tracing studies have revealed that in young‐to‐middle–aged mice, SSCs undergo frequent turnover, during which some SSC clones expand, whereas others disappear, maintaining stem cell density at the population level (Hara et al. 2014; Klein et al. 2010; Nakagawa et al. 2007). This expansion of surviving SSC clones is enabled by the open niche, which lacks an apparent anatomical barrier, allowing extensive SSC migration and dynamic interaction with the surrounding microenvironment that regulates SSC homeostasis (Hara et al. 2014; Kitadate et al. 2019; Klein et al. 2010; Yoshida et al. 2007). However, how SSC clonal expansion and the underlying behaviors of individual SSCs, such as proliferation and migration, change with aging remains largely unknown.

The transplantation assay suggested that although the number of transplantable SSCs decreased with age, they remained detectable when testicular cells from 2‐year‐old mice were transplanted into immature mouse testes (Ryu et al. 2006; Zhang et al. 2006). In contrast, germline stem cells cultured in vitro for over 3 years reportedly continued to proliferate but showed a decline in their ability to regenerate spermatogenesis after transplantation into immature testes. This suggests that although SSCs can retain proliferative activity over a long period, their sperm‐forming ability intrinsically deteriorates with in vitro aging (Kanatsu‐Shinohara et al. 2019). As cell intrinsic changes can be passed down within a clone, it becomes important to investigate age‐related alterations in the differentiation capacity and expansion of SSC clones in physiologically aged testes. This raises two key questions: Does a subset of SSC clones in the testes of physiologically aged mice exhibit reduced sperm formation? Do these SSC clones expand within the testicular open niche?

By employing advanced techniques such as single‐cell RNA sequencing (scRNA‐seq), lineage tracing, and intravital live imaging, which were previously applied to study SSCs in young mice, it is now possible to investigate SSCs comprehensively in physiologically aged mouse testes. These methods enable the evaluation of SSC characteristics in old mouse testes, including state heterogeneity, real‐time single‐cell behaviors, and long‐term clonal fate behavior. In addition, combining scRNA‐seq with lineage tracing also allows for the identification of molecular signatures in heterogeneous SSCs that exhibit distinct fate behaviors.

This study aimed to elucidate the effects of physiological aging on the clonal fate behavior of SSCs within the testicular open niche. Using scRNA‐seq, lineage tracing, and intravital live imaging, we first found that GFRα1^+^ cells in old mice retained SSC function, exhibiting state heterogeneity, accelerated proliferation, and persistent motility. We also discovered that a subset of SSCs that did not clonally form spermatids emerged, which was marked by reduced expression of early growth response 4 (*Egr4*) and *Cops5*. Notably, in old mice, the proportion of non–sperm‐forming SSC clones increased compared to young mice, and these clones expanded along the seminiferous tubules, a phenomenon not observed in the testes of young mice. These findings highlight that SSC clones exhibit functional heterogeneity and expansion, emphasizing the key aspects of stem cell aging within the testicular open niche.

## 2 Results

### 2.1 Aged‐Undifferentiated Spermatogonia Support Spermatogenesis in Most Seminiferous Tubules Despite a Decrease in Spermatid Formation

To capture age‐related changes in testicular function, we first analyzed three age groups of C57BL6J male mice: young mice aged 3–4 months, middle‐aged mice aged 15–20 months, and old mice aged 25–28 months. We observed a significant age‐related decrease in both absolute testis weight and testis weight relative to body weight (Figure 1B and Figure S1A). The density of Sertoli cells per cross section of the seminiferous tubules and the mean diameter of the seminiferous tubules did not differ significantly (Figure S1B–D). Combined with a previous study showing that the total length of seminiferous tubules in the mouse testis does not decrease with age (Nakano et al. 2021), our findings suggest that the overall number of Sertoli cells per testis likely remains unchanged during aging. Furthermore, sections of seminiferous tubules in which germ cells were completely absent, often designated as Sertoli cell–only (SCO) tubules, were rarely observed in the current study, and their frequencies out of the total observed tubules did not change significantly with age (Figure S1E,F). Thus, the density of spermatogenic cells at each stage per tubule section was quantified, excluding SCO tubules, and compared among the young, middle‐aged, and old groups (Figure S1D). We observed a slight decrease in the density of total spermatogonia, encompassing both A_undiff_ and differentiating spermatogonia, from the young to old age groups (Figure 1C). With regard to the subpopulation of spermatogonia, we analyzed the densities of GFRα1^+^ and Rarg^+^ (mostly overlapping with Ngn3^+^) A_undiff_ and c‐Kit^+^‐differentiating spermatogonia (Figure 1A). Section immunohistochemistry results showed a decrease in the densities of GFRα1^+^, Rarg^+^, and c‐Kit^+^ spermatogonia per tubule from the young‐to‐old age groups (Figure 1D–F). Consistent with this finding, we observed a decrease in the density of spermatocytes and spermatids per tubule section with age (Figure 1G,I). Notably, the decline in spermatocyte and spermatid density per tubule was more pronounced in the middle‐to‐old age group than in the young‐to‐middle age group (Figure 1G,I). Dot plots of spermatocyte and spermatid densities in individual tubule sections indicated that their reduction with age might reflect a general decrease across the entire testis rather than being due to severe localized decreases in certain areas (Figure 1H,J). Taken together, these observations suggest that spermatogenesis occurs in most regions of the seminiferous tubules, whereas sperm formation decreases in the testes of old mice.

### 2.2 Maintenance of Transcriptional Heterogeneity in Aged Undifferentiated Spermatogonia

**FIGURE 2:**
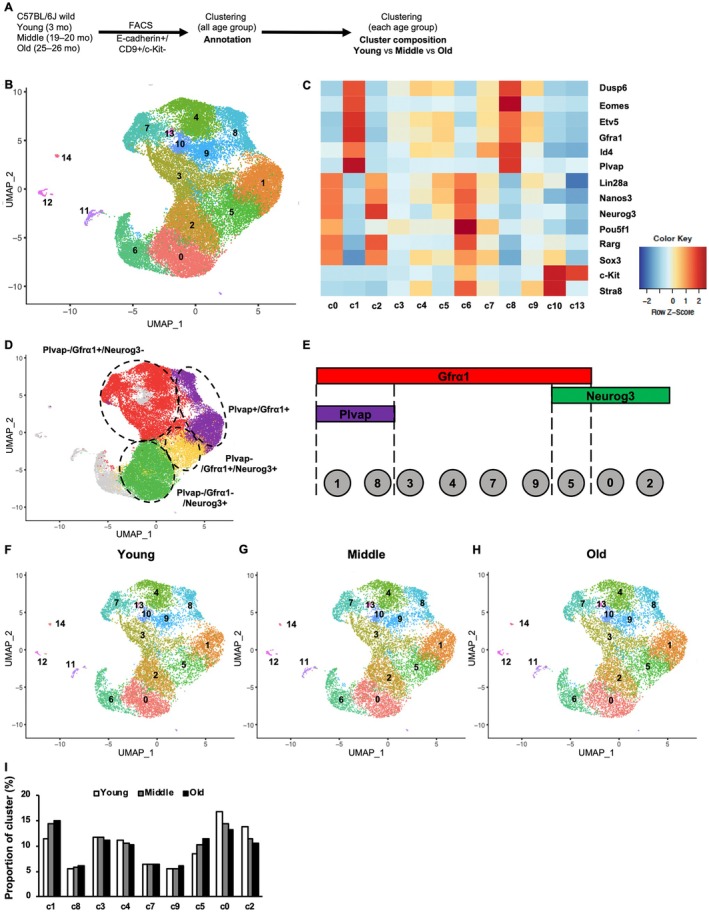
Age‐related changes in heterogeneity of gene expression in the undifferentiated spermatogonia populations. (A) Experimental single‐cell RNA sequencing workflow for the undifferentiated spermatogonia (A_undiff_) cells. E‐cadherin^+^/CD9^+^/c‐Kit^−^ cells were sorted using FACS from C57BL/6J wild‐type mice at different ages: young (3 months), middle (19–20 months), and old (25–26 months). The number of individuals used per age group was *n* = 2. (B) UMAP plot showing all age groups combined (*n* = 6). Different colors and numbers indicate distinct clusters. (C) Heat map displaying marker gene expression across clusters, with mRNA levels represented by Z‐scores. (D) UMAP plot showing all age groups (*n* = 6), with clusters assigned based on the expression of known marker genes representing different states of A_undiff_ cells. (E) Schematic representation of the classification of each cluster into distinct A_undiff_ states, based on data from panels (C) and (D). (F–H) UMAP plots for each age group: (F) Young (*n* = 2), (G) middle (*n* = 2), and (H) old (*n* = 2). (I) Comparison of the proportion of cells in each cluster of the total A_undiff_ cells across age groups.

To investigate age‐associated changes in the gene expression heterogeneity of A_undiff_, scRNA‐seq analysis was conducted on the population of A_undiff_ prepared through sorting of the testicular cells as E‐cadherin^+^/CD9^+^/c‐Kit^−^ fractions, as previously described (Nakagawa et al. 2021) (Figure 2A and Figure S2). Dimension reduction analysis was performed for all data collected from the different age groups, which identified 15 distinct clusters (Figure 2B). In the absence of the pan‐germ cell marker *Ddx4*, clusters 11, 12, and 14 appeared to contain contaminated somatic cells (Figure S3A). Within the *Ddx4*‐expressing clusters, clusters 0, 1, 2, 3, 4, 5, 7, 8, and 9 could be annotated as A_undiff_, based on the expression of multiple marker genes (*Dusp6*, *Eomes*, *Etv5*, *Gfrα1*, *Id4*, *Plvap*, *Lin28*, *Nanos3*, *Ngn3*, *Pou5f1*, *Rarg*, and *Sox3*), whereas clusters 6, 10, and 13 could represent differentiating spermatogonia based on their high *c‐Kit* expression levels (Figure 2B,C). Expression of key A_undiff_ marker genes (*Plvap*, *Gfrα1*, and *Ngn3*) in young testes indicated the following distinct subpopulations of A_undiff_: *Plvap* ^+^/*Gfrα1* ^+^/*Ngn3* ^−^ in Clusters 1 and 8, *Plvap* ^−^/*Gfrα1* ^+^/*Ngn3* ^−^ in Clusters 3, 4, 7, and 9, *Plvap* ^−^/*Gfrα1* ^+^/*Ngn3* ^+^ in Cluster 5, and *Plvap* ^−^/*Gfrα1* ^−^/*Ngn3* ^+^ in Clusters 0 and 2 (Figure 2B–D) (Nakagawa et al. 2021). Thus, we annotated the cell state order toward differentiation within A_undiff_ as follows: Cluster 1/8 → Cluster 3/4/7/9 → Cluster 5 → Cluster 0/2 (Figure 2D,E). This was also supported by the principal component analysis (PCA) and hierarchical cluster analysis using differentially expressed genes in each cell population: *Plvap* ^+^/*Gfrα1* ^+^/*Sox3* ^−^/*Ngn3* ^−^ (201 genes), *Plvap* ^−^/*Gfrα1* ^+^/*Sox3* ^−^/*Ngn3* ^−^ (56 genes), *Plvap* ^−^/*Gfrα1* ^+^/*Sox3* ^+^/*Ngn3* ^−^ (246 genes), and *Plvap* ^−^/*Gfrα1* ^−^/*Sox3* ^−^/*Ngn3* ^+^ (525 genes) (Figure S3B,C) (Nakagawa et al. 2021). We confirmed that all clusters were consistently present across individuals in each age group (Figure S3D). Notably, the consistent presence of all A_undiff_ clusters (0, 1, 2, 3, 4, 5, 7, 8, and 9) across all age groups, combined with the minimal differences in the composition ratios of these clusters, suggests that the transcriptional heterogeneity of A_undiff_ is largely preserved across different ages (Figure 2F–I).

### 2.3 Maintenance of Aged GFRα1 ^+^ Spermatogonia at the Population Level in the Testes of Old Mice

**FIGURE 3:**
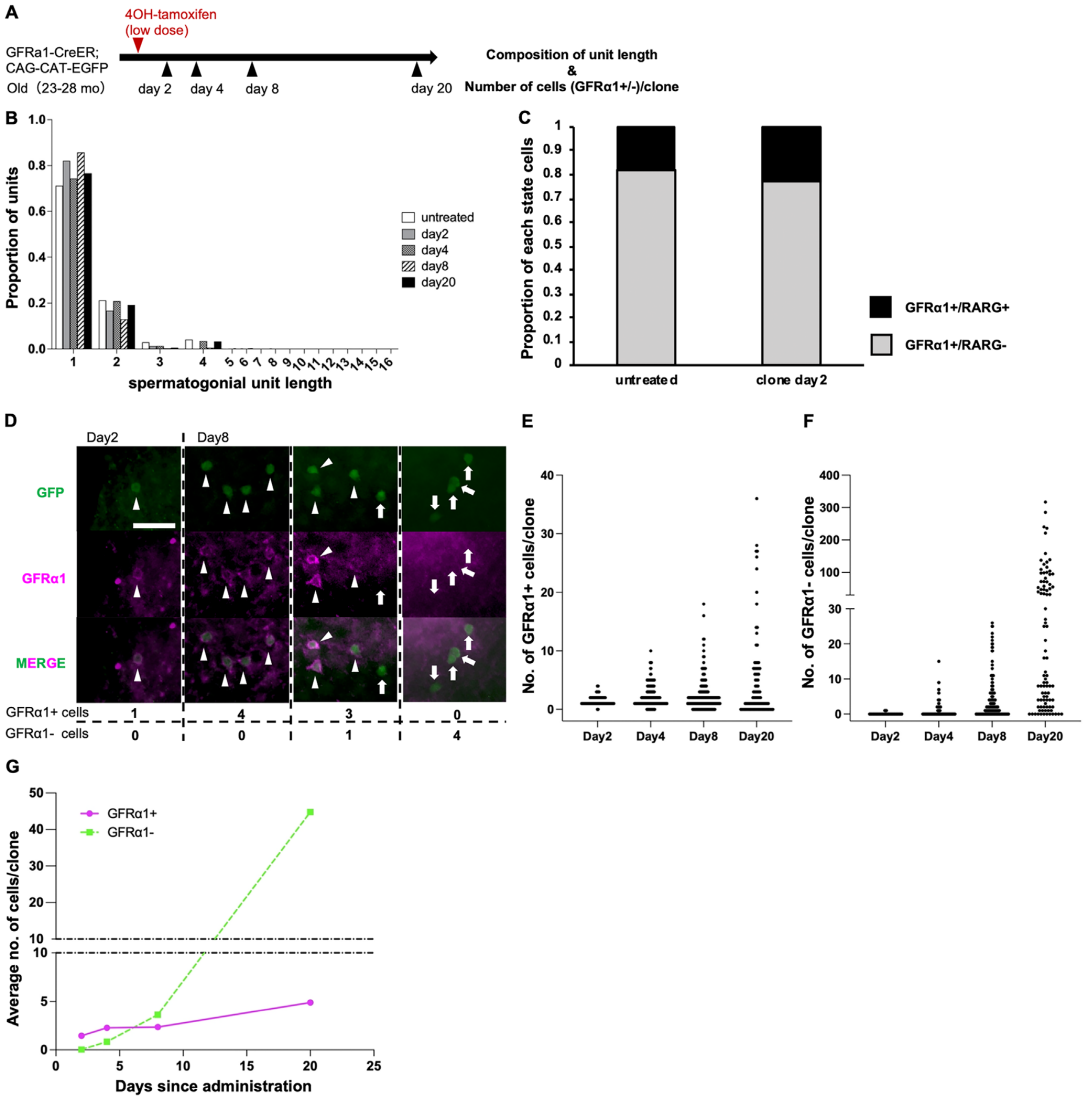
Short‐term clonal fate analysis of GFRα1^+^ cells in old mice. (A) Experimental schedule for the clonal fate analysis of pulse‐labeled GFRα1^+^ units in (B–G). *GFRα1‐CreER* ^*T2*^ and *CAG‐CAT‐EGFP* mice (23–28 months) were administrated 0.25 mg 4OH‐tamoxifen, to sparsely label the GFRα1^+^ spermatogonia, and analyzed at the indicated time points. The number of individuals used at each time point was as follows: *n* = 3, 4, 3, and 2 (2, 4, 8, and 20 days post induction, respectively). Ages refer to when the testes were harvested. (B) Composition of the unit length of GFRα1^+^ cells in 23 months wild‐type mice (*n* = 3) and labeled GFRα1^+^ cells across total clones over time. (C) Proportion of cells classified into two differentiation stages (GFRα1^+^/RARG^−^, GFRα1^+^/RARG^+^) of untreated GFRα1^+^ cells from 23 months wild‐type mice (*n* = 3) and labeled GFRα1^+^ cells at 2 days post induction. (D) Whole‐mount GFP (green) and GFRα1 (magenta) staining image of a seminiferous tubule, with examples of clones at 2 and 8 days post induction. The lower table shows the counted cell numbers. Scale bar, 50 μm. Arrowheads indicate GFP^+^/GFRα1^+^ cells and arrows the GFP^+^/GFRα1^−^ cells. (E, F) Distribution of clone size, as measured by the number of (E) GFRα1^+^ and (F) GFRα1^−^ cells per clone over time. Each dot indicates one clone. The number of clones at Days 2, 4, 8, and 20 were 63, 135, 178, and 91, respectively. (G) Average number of GFRα1^+^ (solid line) and GFRα1^−^ (dotted line) cells per clone over time. Syncytia of 32 or more cells, all of which were GFRα1^−^ and observed 20 days after the pulse, were scored as 32‐cell syncytia because of the difficulty in making a precise count; this method underestimates the number of GFRα1^−^ cells (dotted line).

The presence of common subclusters of GFRα1^+^ spermatogonia between young and aged mouse testes led to a hypothesis that GFRα1^+^ spermatogonia might function as SSCs in the testes of old mice, as seen in young mice. To test this hypothesis, we conducted clonal fate analysis of GFRα1^+^ cells for 20 days (the duration during which we could trace all surviving differentiating progenitor cells before sperm release) via a low‐dose single administration of 4OH‐tamoxifen to old‐age *GFRα1‐CreER;CAG‐CAT‐EGFP* mice (Hara et al. 2014; Kawamoto et al. 2000) (Figure 3A). A comparable experiment had previously been performed in young mice, where most labeled clones initially contained a single GFRα1^+^ cell. Over a period of 2–20 days, these clones exhibited significant variability in their fates; however, the average number of GFRα1^+^ cells per clone remained stable. This suggests that GFRα1^+^ cells in young mouse testes are maintained at the population level (Hara et al. 2014).

In our study of old mice, we observed consistent clonal fate behavior in young mice. Given the minimal decrease in the density of GFRα1^+^ spermatogonia from the young‐to‐old age groups (2.17% average decrease per month), we assumed that the dynamics of GFRα1^+^ spermatogonia in old mice are under a quasi‐steady state (Figure 1D). Shortly (2 days) after 4OH‐tamoxifen administration, the majority of GFP‐labeled clones contained a single GFRα1^+^ A_s_ (cell number = 1) and less frequent A_pr_ (cell number = 2) and A_al_ (cell number = three or more). Each of these—A_s_, A_pr_, and A_al_—is referred to as a single “unit” (Figure 3B). Among these GFRα1^+^‐labeled cells, the proportion of Rarg^+^ cells was comparable to that observed among GFRα1^+^ cells in untreated tissue (Figure 3C). These data suggest that the labeled cell population can be deemed representative of the entire GFRα1^+^ cell population in the testes of old mice. Then, we found that clones derived from a labeled cell exhibited variable fates in terms of the number of GFRα1^+^ and GFRα1^−^ cells within a clone at Day 8 post labeling, which became more divergent by Day 20 (Figure 3D–F and Table S1). By contrast, the average number of GFRα1^+^ cells per clone was largely maintained while producing GFRα1^−^‐differentiating progeny over a period of 20 days (Figure 3G). The average unit composition of GFRα1^+^ cells in clones in old‐age mice remained constant for 20 days, similar to that of the untreated GFRα1^+^ unit composition, validating the maintenance of the GFRα1^+^ population over time (Figure 3B). These results indicate that the initially labeled GFRα1^+^ population continually generated largely the same number of GFRα1^+^ spermatogonia, with consistent unit composition, and gave rise to GFRα1^−^‐differentiating cells in old mice, similar to what was observed in young mice.

### 2.4 Proliferation, Survival, and Migration of Aged GFRα1 ^+^ Spermatogonia on the Basement Membrane

**FIGURE 4:**
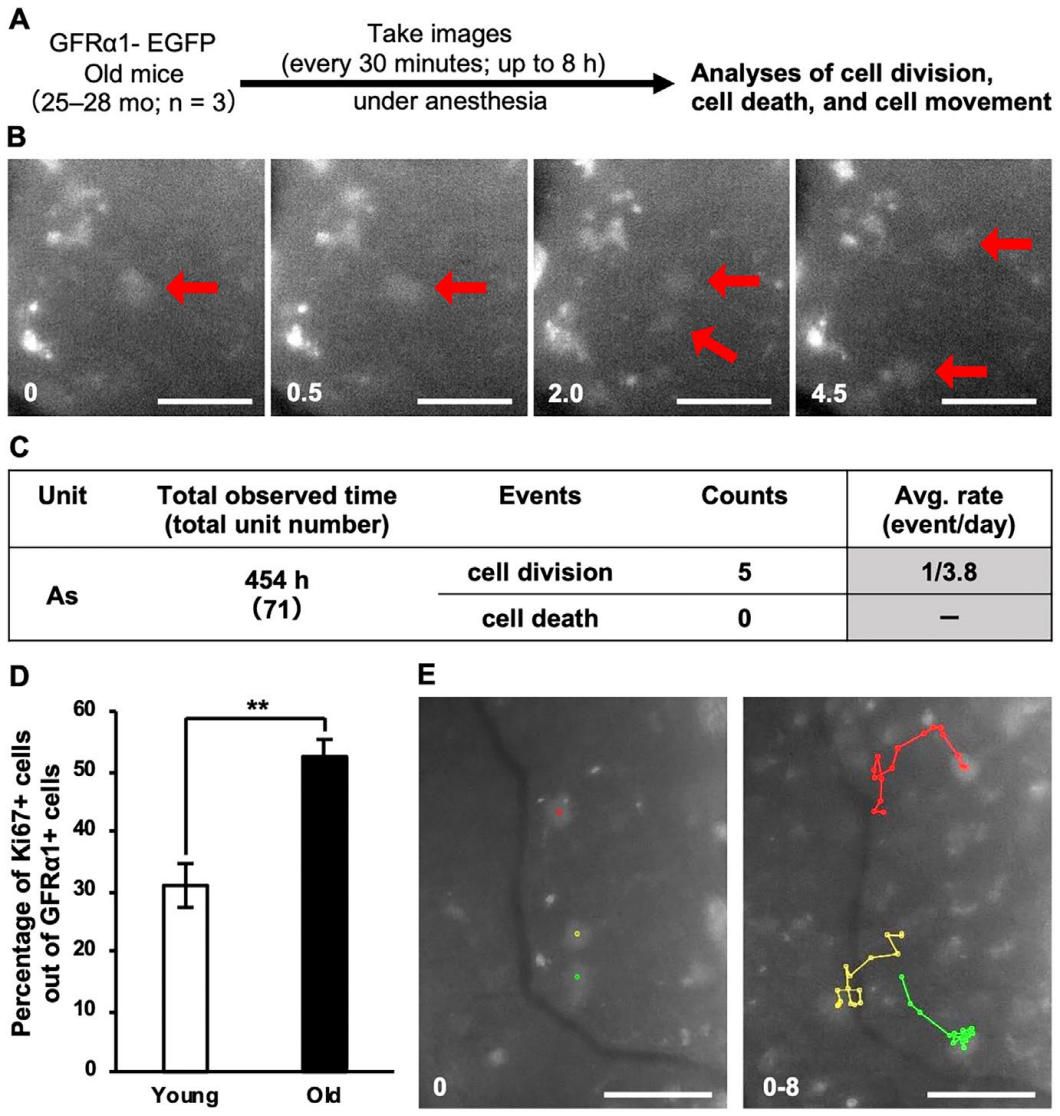
Live imaging of GFRα1^+^ spermatogonia in old mouse testes. (A) Experimental schedule for live imaging of GFRa1‐EGFP knock‐in old mouse testes. (B) Example of cell division. Arrows indicate GFRα1‐EGFP^+^ spermatogonia and numerals the time elapsed since the start of the observation (hours). (C) Table showing the cell division and death of GFRα1‐EGFP^+^ A_s_ spermatogonia during the total observation period. Average rates of cell division were calculated as “counts of cell division”/“total observed time.” (D) Percentage of Ki67^+^ cells among GFRα1^+^ cells in C57BL/6J wild‐type mice. Young, 2 months (*n* = 3) and old, 22 months (*n* = 3). Averages ± SD, **; *p* < 0.01 (Student's *t*‐test). The number of sections and the number of GFRα1^+^ cells for each individual were as follows (number of sections/number of GFRα1^+^ cells): young, (2/191, 2/121, 2/186) and old, (2/223, 2/293, 2/276). (E) Trajectories of individual GFRα1‐EGFP^+^ A_s_ spermatogonium over 8 h of observation, shown in different colors. (B, E) Scale bars, 50 μm.

As maintenance of the GFRα1^+^ cell density is supported by the proliferation, survival, and migration of these cells, we then evaluated GFRα1^+^ cell behavior by performing intravital live imaging of the aged testes of old (25–28 months), GFRα1‐EGFP mice for up to 8 h (Figure 4A). A comparable live imaging experiment had previously been reported in young mice, where GFRα1‐EGFP^+^ cells migrated, divided once every 10 days, and died infrequently (Hara et al. 2014).

We found that the averaged frequency of cell division for GFRα1‐EGFP^+^ A_s_ cells was once per 3.8 days (five times for 454 h), which was higher than that in young mice (Figure 4B,C and Video S1) (Hara et al. 2014). Furthermore, in wild‐type mice, the proportion of Ki67^+^ cells among total GFRα1^+^ cells was significantly higher in old mice than that in young mice, reinforcing the accelerated proliferation of GFRα1^+^ cells with age (Figure 4D). We did not observe cell death for GFRα1‐EGFP^+^ A_s_ cells during the observation period (454 h), similar to what was previously observed in young mice (Hara et al. 2014) (Figure 4C). Live‐imaging analysis also showed the migration of GFRα1‐EGFP^+^ A_s_ cells on the basement membrane near vasculature, consistent with observations in young mice (Figure 4E and Video S2) (Hara et al. 2014). These observations suggest that GFRα1^+^ cells in the testes of old mice exhibit accelerated proliferation, without reducing their survivability and motility, during their maintenance.

### 2.5 Long‐Term Contribution of GFRα1 ^+^ Cells to Spermatogenesis in Aged Testes

**FIGURE 5:**
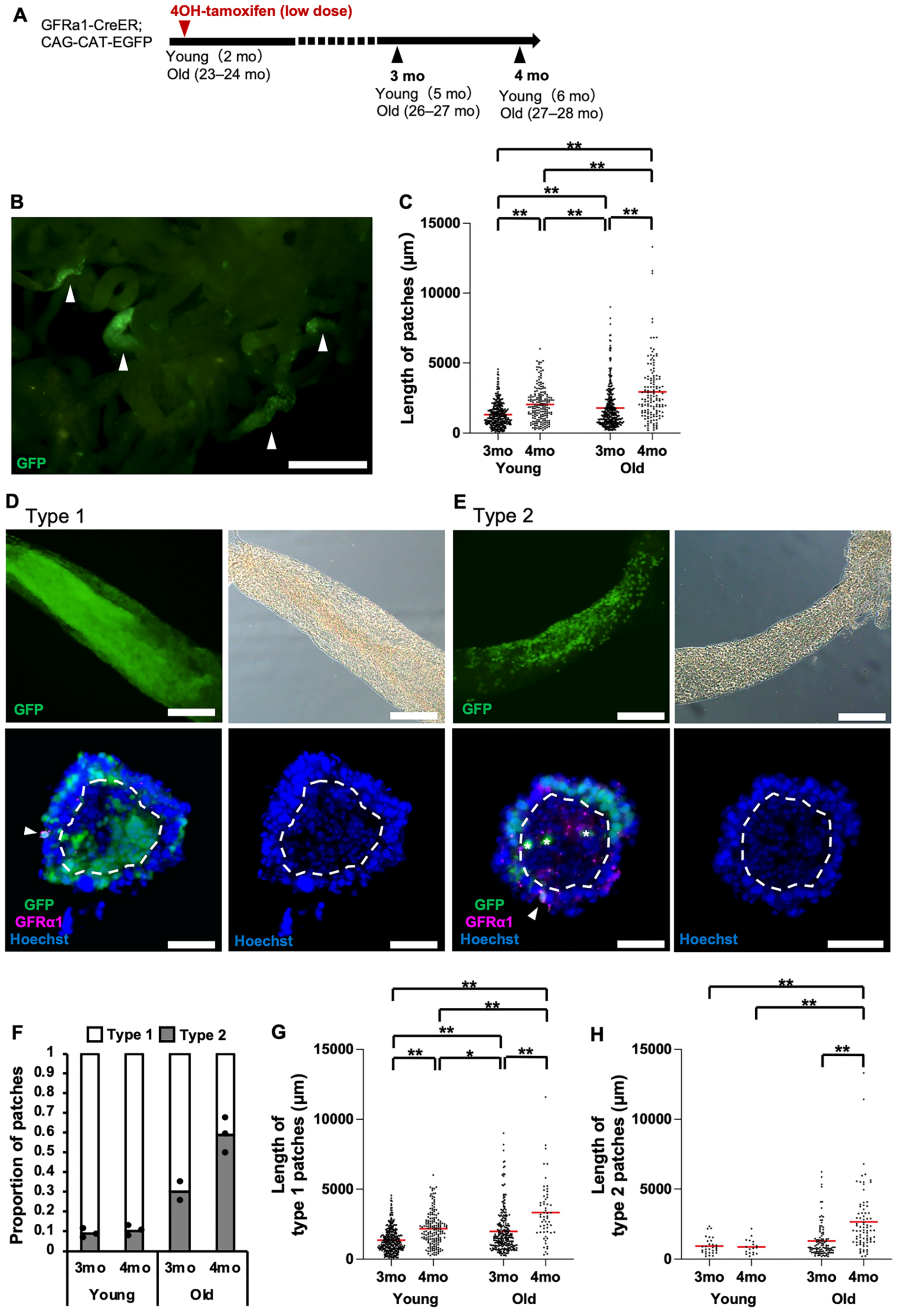
Long‐term dynamics of GFRα1^+^ cell–derived clones in aged mice. (A) Experimental schedule for clonal fate analysis of pulse‐labeled GFRα1^+^ units in (B–H). *GFRα1‐CreER* ^*T2*^ and *CAG‐CAT‐EGFP* mice (2 and 23–24 months, respectively) were administrated 0.25 mg 4OH‐tamoxifen to sparsely label GFRα1^+^ spermatogonia. Clonal analysis, including the evaluation of patch length and classification of clone types, was performed at the indicated time points. The number of individuals (and patches) used at each time point was as follows: young, *n* = 3 (341), 3 (184); old, *n* = 2 (356), 3 (139) (3 and 4 months post induction, respectively). (B) Seminiferous tubules at 3 months post induction, showing GFP^+^ clonal patches (arrowheads). Scale bar, 1 mm. (C) Distribution of clonal patch lengths at 3 and 4 months post induction for young and old mice. (D, E) Upper panels: whole‐mount GFP staining of clonal patches (left) and differential interference contrast micrographs (right) at 3 months post induction for old mice, showing Type 1 (D) and Type 2 (E) clones. Lower panels: Cross‐sectional staining of clonal patches with GFP and Hoechst (left) and Hoechst (right) at 3 months post induction for old mice, showing Type 1 (D) and Type 2 (E) clones. Cross‐sectional staining was performed on the samples after they were distinguished into Type 1 and Type 2 clones via whole‐mount staining. Arrowheads, GFRα1^+^ cells; region inside the dotted line, area where spermatids are found; *, GFP^+^/Hoechst^−^ object; scale bars, 200 μm. (F) Proportion of clonal patches at 3 and 4 months post induction for young and old mice. Black dots indicate the proportion of Type 2 patches relative to the total number of patches in each testis. Boundaries of the stacked graphs indicate the mean values for each group. All patches were classified as Type 1 or Type 2. (G, H) Distribution of Type 1 (G) and Type 2 (H) clonal patch lengths at 3 and 4 months post induction for young and old mice. (C, G, H) *p < 0.05; **p < 0.01 (Dunn's multiple comparison test).

To evaluate sperm formation by the individual GFRα1^+^ cells of old mice compared to that in young mice, GFP^+^ cell clones originating from individual GFRα1^+^ cells were analyzed at 3 and 4 months after a single administration of 4OH‐tamoxifen to young and old *GFRα1‐CreER; CAG‐CAT‐EGFP* mice (Hara et al. 2014) (Figure 5A). In young mice, clones derived from the pulse‐labeled, single GFRα1^+^ cells were reported to evolve into large patches containing SSCs and differentiating progenies over a few months (Hara et al. 2014). In old‐age testes, GFP^+^ clonal patches, originating from a single GFRα1^+^ cell, were observed 3 months after administration, suggesting the long‐term contribution of GFRα1^+^ cells to spermatogenesis during old age (Figure 5B). A significant extension of patch length was observed from 3 to 4 months post labeling, and its distribution broadened in young mouse testes (Figure 5C). This clonal territorial expansion and broadening was also observed in the testes of old mice (Figure 5C). However, the average patch length was significantly longer in old mouse testes than in young testes at both 3 and 4 months after treatment (Figure 5C). These findings suggest that clones originating from a single GFRα1^+^ cell contribute to long‐term spermatogenesis during old age and expand more quickly in older testes than in younger ones.

### 2.6 Clonal Expansion of Aged SSCs If Not Producing Sperm

We evaluated the distribution and differentiation stage of the GFP^+^ cells in each clone by observing whole‐mount seminiferous tubules. We found that GFP^+^ clones could be classified into two types: those with GFP^+^ cells in all or part of the adluminal region (Type 1) and those restricted only to the surface region of the tubules (Type 2) (Figure 5D,E; upper panels). Examination of tubule cross sections confirmed that the Type 1 GFP^+^ patch spanned from GFP^+^/GFRα1^+^ spermatogonia to GFP^+^ spermatids (patch number = 69). In contrast, the Type 2 GFP^+^ patch contained GFP^+^/GFRα1^+^ spermatogonia, GFP^+^/GFRα1^−^ spermatogonia, and GFP^+^ spermatocytes, which were arrested at the pachytene stage, whereas GFP^+^ spermatids were not observed (patch number = 31) (Figure 5D,E [lower panels] and Figure S4A). In the Type 2 patch section, abundant GFP^−^ spermatids were observed within the luminal region of the same seminiferous tubule section (Figure 5E [lower panel] and Figure S4A). These findings suggest the presence of GFRα1^+^ cell–derived clones without sperm formation in mouse testes.

We then focused on the clonal fate behavior of GFRα1^+^ cells in Type 1 and Type 2 patches in the young and old age groups. The proportion of Type 2 GFP^+^ patches out of the total GFP^+^ patches was higher in older mice than in younger mice, and this proportion increased between 3 and 4 months post labeling, specifically in the old‐age group (Figure 5F). In Type 1 GFP^+^ patches, the average length increased from 3 to 4 months post labeling and the distribution became broader in both young and old testes (Figure 5G). Notably, the average length of Type 1 GFP^+^ patches was significantly longer, and their lengths were more widely distributed in old testes than in young testes at both 3 and 4 months post labeling (Figure 5G). In Type 2 GFP^+^ patches, their average length increased and their distributions became broader between 3 and 4 months post labeling in old testes, whereas they remained short and unchanged in young testes (Figure 5H). The average length of Type 2 GFP^+^ patches was significantly longer, and their lengths were more widely distributed in old testes than in young testes at 4 months post labeling (Figure 5H). These data suggest that in old mice, the proportion of non–sperm‐forming SSC clones increases relative to the total SSC clones, and these non–sperm‐forming SSC clones expand along the seminiferous tubules, a phenomenon not observed in young mice.

### 2.7 Identification of the Molecular Signature of Non–Sperm‐Forming SSCs

**FIGURE 6:**
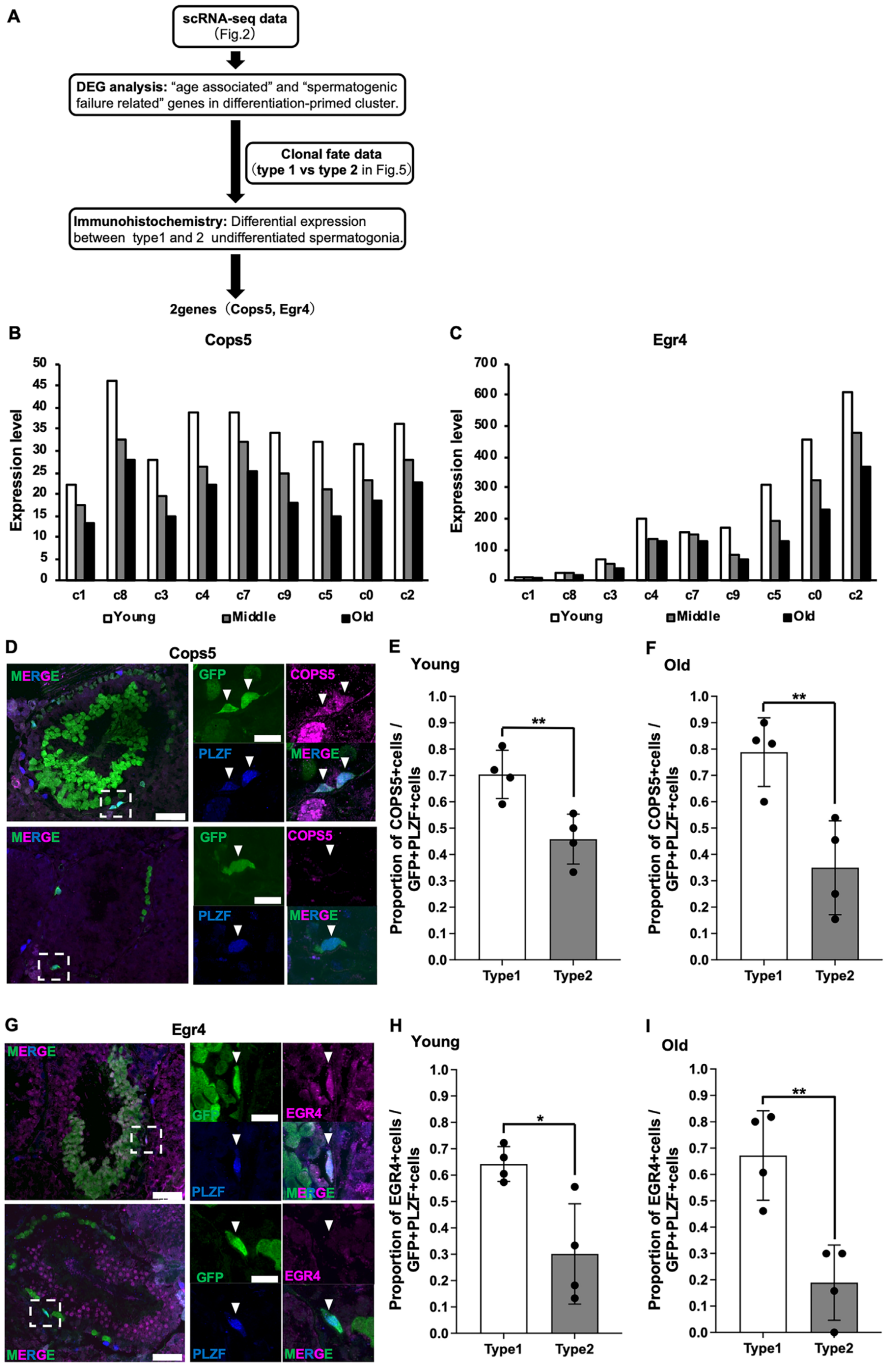
(A) Screening for differentially expressed genes associated with the functional decline of spermatogonial stem cells (SSCs). Workflow for screening differentially expressed genes between functionally competent SSCs in Type 1 patches and functionally declined SSCs in Type 2 patches using single‐cell RNA sequencing (scRNA‐seq) and clonal fate data. (B, C) Comparison of *Cops5* (B) and *Egr4* (C) expression levels in each undifferentiated spermatogonia (A_undiff_) cluster from young, middle‐aged, and old mice, based on the scRNA‐seq data (see Figure 2). (D) Left: cross‐sectional GFP (green), COPS5 (magenta), and PLZF (blue) staining in Type 1 (upper) and Type 2 (lower) clones at 4 months post induction for old mice. Scale bars, 50 μm. Right: enlarged view of the dotted box indicated in the left image (upper left, GFP; upper right, COPS5; lower left, PLZF; lower right, merge). Arrowheads, PLZF^+^ cells; scale bars, 15 μm. (E, F) Proportion of cells expressing COPS5 out of the PLZF^+^ cells in Type 1 and Type 2 GFP^+^ cells at 3 (*n* = 2) and 4 months (*n* = 2) post induction for young (E) and old (F) mice. The number of PLZF^+^GFP^+^ cells counted for each mouse at 3 months post induction was (E) Type 1: 22, 16 and Type 2: 9, 9 and (F) Type 1: 18, 15 and Type 2: 13, 13, whereas that at 4 months post induction was (E) Type 1: 43, 65 and Type 2: 9, 10 and (F) Type 1: 30, 39 and Type 2: 12, 11. Black dots indicate the proportion of cells expressing COPS5 out of the PLZF^+^ cells in each testis. (G) Left: cross‐sectional GFP (green), EGR4 (magenta), and PLZF (blue) staining in Type 1 (upper) and Type 2 (lower) clones at 4 months post induction for old mice. Scale bars, 50 μm. Right: enlarged view of the dotted box indicated in the left image (upper left, GFP; upper right, EGR4; lower left, PLZF; lower right, merge). Arrowheads, PLZF^+^ cells; scale bars, 15 μm. (H, I) Proportion of cells expressing EGR4 among the PLZF^+^ cells in Type 1 and Type 2 GFP^+^ cells at 3 (*n* = 2) and 4 months (*n* = 2) post induction for young (H) and old (I) mice. The number of PLZF^+^GFP^+^ cells counted for each mouse at 3 months post induction was (H) Type 1: 15, 18 and Type 2: 9, 11 and (I) Type 1: 15, 13 and Type 2: 9, 19, whereas that at 4 months post induction was (H) Type 1: 75, 86 and Type 2: 9, 15 and (I) Type 1: 33, 28 and Type 2: 10, 10. Values represent averages ± SD; **P < 0.01, *P < 0.05 (Student's *t*‐test). Graphs and statistical analyses are based on four testes 3 and 4 months post induction. Black dots indicate the proportion of cells expressing EGR4 out of the PLZF^+^ cells in each testis.

To explore the molecular signature associated with the observed decline in sperm formation in SSCs, we conducted an integrated screening combining scRNA‐seq and long‐term lineage tracing (Figure 6A). Initially, we assumed that SSC differentiation failure was primarily linked to alterations in gene expression in the differentiation‐primed state of SSCs. Therefore, we focused on the gene expression profiles of *Plvap* ^−^/*Gfrα1* ^+^/*Ngn3* ^+^ A_undiff_ (Cluster 5 in Figure 2) from the scRNA‐seq data, identifying 49 genes that were downregulated with aging within this cluster, whereas no genes showed a significant increase in expression (Table S2). Among the 49 downregulated genes, we identified *Cops5* and *Egr4* as genes whose knockout led to spermatogenic abnormalities, based on previous reports (Hogarth et al. 2010; Huang et al. 2020; Tourtellotte et al. 1999). The expression of these genes was reduced in all A_undiff_ clusters in the testes of old mice compared to that in young mice (Figure 6B,C). Furthermore, the distribution of *Cops5* and *Egr4* expression levels in individual cells in each cluster was compared among different age groups, and the distribution was divided into two groups, high and low expression, in all clusters of all age groups. The mode of high expression did not change between the three age groups; however, the proportion of cells with low expression increased. This suggests that gene expression did not decrease uniformly in all cells; rather, the proportion of cells with reduced expression increased (Figure S5A,B). In the immunohistochemical analyses of Type 1 and Type 2 clonal patches, the proportion of A_undiff_ expressing either COPS5 or EGR4 was significantly lower in Type 2 patches than in Type 1 patches in both young and old mice (Figure 6D–I). These findings suggest that reduced expression of COPS5 and EGR4 in the A_undiff_ of Type 2 patches is associated with incomplete spermatogenesis, regardless of whether clonal expansion occurs.

## 3 Discussion

Advancements in analytical technologies, including scRNA‐seq, lineage tracing, and intravital live imaging, have allowed us to evaluate the clonal fate of SSCs in physiologically aged mice. Our study revealed that while the basic principles governing SSC maintenance remain consistent throughout life, age‐specific changes in SSC clonal fate behavior within the testicular open niche likely contribute to decreased sperm productivity and genetic diversity.

### 3.1 Maintenance of GFRα1 ^+^ Cells and Age‐Related Changes

We observed that GFRα1^+^ spermatogonia are maintained on the basement membrane in the testes of 2‐year‐old mice in a quasi‐steady state, although their density decreased slowly from a young age. Clonal fate analyses over a 20‐day period suggested that morphologically heterogeneous GFRα1^+^ cells—including mostly A_s_, A_pr_, and a few A_al_ cells—are largely maintained at the population level, whereas individual clones follow various fate behaviors, as observed in young mice (Hara et al. 2014; Klein et al. 2010; Nakagawa et al. 2007). We also found that A_undiff_ largely maintained gene expression heterogeneity with no apparent loss or gain in the subpopulations. Consistent with a previous report showing increased proliferation of A_undiff_ in aged rats (Kanatsu‐Shinohara et al. 2019), we found that GFRα1^+^ cells from aged mice proliferated at a higher frequency than those from younger mice did. This may contribute to sustaining sperm productivity to some extent, despite the reduced density of GFRα1^+^ cells compared to that in young mice. The fact that the expansion of the average patch length of clones (both Type 1 and Type 2) was faster in old than in young testes supports the possibility that frequent SSC turnover occurs in old testes due to increased proliferation of GFRα1^+^ cells.

Live imaging revealed the high survival and sustained motility of GFRα1^+^ cells on the basement membrane of old mouse testis. The motility of GFRα1^+^ cells was further validated by the territorial clonal expansion of SSCs observed along the seminiferous tubules over the long term. This is an important finding because GFRα1^+^ cell motility is known to be essential for the replacement of SSCs to maintain their local density during steady‐state spermatogenesis in young mice (Hara et al. 2014; Kanamori et al. 2019; Kitadate et al. 2019; Klein et al. 2010). Despite reports of thickening and folding of the testicular basement membrane with age, possibly due to aberrant turnover in aged testes (Gosden et al. 1982; Richardson et al. 1995), our data indicate that age‐related morphological changes have minimal effects on spermatogonial survival and migration. These findings suggest the presence of robust regulatory mechanisms that ensure high survival and persistent migration even on the basement membrane in an aged testicular open niche environment, facilitating the sustained territorial evolution of SSCs during aging.

### 3.2 Emergence and Expansion of Non–Sperm‐Forming SSC Clones in Old Mouse Testis

We identified the emergence of SSC clones that did not form sperm, referred to as non–sperm‐forming SSC clones, in mouse testes. In Type 2 clones, none of the SSCs produced spermatids as spermatogenesis was arrested at the pachytene spermatocyte stage. Given that these clones are expected to contain many GFRα1^+^ cells, it is unlikely that all of them failed to differentiate during this period is merely coincidental. Moreover, the surrounding functional SSCs differentiated properly in the same local environment within seminiferous tubules. These observations suggest that defective spermatogenesis in Type 2 clones is induced by intrinsic regulation within SSCs, rather than by external factors from the testicular niche environment. Therefore, we hypothesized that the molecular signatures of non–sperm‐forming SSCs related to their non–sperm‐forming characteristics would be present. By confirming this, we found that a reduction in EGR4 (early growth response 4) and COPS5 (subunit 5 of the COP9 signalosome complex) expression could serve as signatures of non–sperm‐forming SSCs. Loss of the transcription factor Egr4 has been reported to block spermatogenesis at the early mid pachytene spermatocyte stage, and the loss of Cops5 in spermatogonia leads to spermatogenic failure around the spermatocyte stage (Hogarth et al. 2010; Huang et al. 2020; Tourtellotte et al. 1999).

Although these changes in gene expression were observed in A_undiff_ cells within non–sperm‐forming SSC clones in both young and old mice, their long‐term behaviors differed between the two age groups. First, the proportion of non–sperm‐forming SSC clones among the total SSC clones was higher in the old age group than that in the young age group. Second, the proportion of non–sperm‐forming SSC clones among the total SSC clones increased in the old age group but not in the young age group. Third, the average length of non–sperm‐forming SSC clones was greater in the old age group than that in the young age group. Finally, non–sperm‐forming SSC clones expanded in old age and remained constant in the young age group. Given these differences, the effect of non–sperm‐forming SSCs on testicular sperm productivity is expected to be greater in old mice than in young mice. Importantly, these non–sperm‐forming SSC clones may act as “space occupiers” within the testicular open niche, potentially restricting the niche space available for functional SSCs. Therefore, this age‐specific spatial competition could further reduce the overall sperm output in old mice.

The reduced expression of Egr4 and Cops5 was observed in A_undiff_ cells within non–sperm‐forming SSC clones in both young and old mice. These changes alone are insufficient to explain the old‐age‐specific expansion of Type 2 SSC clones, suggesting the involvement of additional mechanisms unique to the aged testis. The old‐age–specific expansion of Type 2 SSC clones may be driven either by intrinsic, cell‐autonomous factors within aged SSCs or by a combination of intrinsic factors within aged SSCs and extrinsic influences from the aged testicular environment, such as age‐related changes in Sertoli cells (Gosden et al. 1982; Tanemura et al. 1994; Zhang et al. 2023). Further studies are required to elucidate the mechanisms responsible for the old‐age–specific expansion of Type 2 SSC clones.

Although we categorized Type 1 and Type 2 patches based on the presence or absence of spermatids, we could not rule out additional minor behavioral heterogeneity within each clone type due to aging‐related complexities, such as localized severe tissue damage. Additionally, in our long‐term clonal fate analysis, as spermatogenic cells are released as sperm from the seminiferous epithelium approximately 35 days after SSC differentiation begins (Russell et al. 1990), we were only able to assess SSC differentiation that occurred within the past month at the time of analysis. Therefore, it remains unclear whether the observed functional decline in SSC clones is irreversible over longer periods and how it might affect sperm production in the epididymis. Given that nonrandom and fluctuating contributions of SSC clones to offsprings have been reported following transplantation (Kanatsu‐Shinohara et al. 2016), future studies are needed to clarify whether Type 1 and Type 2 SSC clones interconvert or remain distinct over time in aged mouse testis.

### 3.3 Silent SSC Functional Decline in Old‐Age Testes

As previously discussed, we observed that, in the open niche of the testis, non–sperm‐forming SSCs can proliferate and migrate along the basement membrane. This migration leads to expansion of their clonal territory on the basement membrane, where they are unable to produce sperm. This phenomenon potentially hinders spermatogenesis by occupying the niche area of the testes via non–sperm‐forming SSCs. However, in the present histological study, spermatid‐depleted seminiferous tubules were rarely observed. Instead, GFP^−^ spermatids originating from the surrounding GFP^−^ SSCs were predominantly localized in the apical region of the seminiferous epithelium within these clone patches. During spermatogenesis, there is a well‐known mechanism for adjusting the density of differentiating cells through their translocation and death (De Rooij and Lok 1987; Klein et al. 2010; Yoshida et al. 2007). Therefore, the impairment of spermatogenesis within seminiferous tubules due to non–sperm‐forming SSCs may be mitigated by the compensatory production of spermatids by the surrounding sperm‐forming SSCs. Taken together, spatial intermingling of functionally heterogeneous SSC clones may mitigate the adverse effects of SSC aging on sperm production, thereby ensuring reduced but continuous spermatogenesis along seminiferous tubules despite aging. On the basis of these observations, we propose that the phenomenon of “silent SSC functional decline” progresses even in aged testes, where germ cells are not completely depleted.

### 3.4 Types of Tissue Environments and Age‐Related Behavioral Changes of Stem Cells

This study highlights how the tissue environment influences the aging process of stem cells and their progeny. In closed niches, such as those of intestinal and hair follicle stem cells in mammals, where the anatomical space is limited, the impact of functionally declining stem cell clones with physiological aging is expected to be confined to isolated niche environments. In contrast, open niches, such as those for hematopoietic stem cells (HSCs) and SSCs in mammals, allow stem cells to exert the influence of their clonal function over larger areas, potentially amplifying the negative effects of functional decline on tissue integrity. Therefore, understanding the spatial and temporal dynamics of aged stem cell clones within open niches is crucial for understanding the impact of stem cell aging on open‐niche–based tissues. Although behavioral fate changes in HSCs during their differentiation have been reported to occur in their open niche, the spatial distribution and expansion of these stem cell clones within tissues remain uncertain because of the intermingling of hematopoietic cells within the bone marrow (Grover et al. 2016; Kowalczyk et al. 2015; Poscablo et al. 2024). Because SSCs are localized within a nearly two‐dimensional and continuous open field of the seminiferous epithelium, the clonal expansion of aged SSCs, regardless of their differentiation fate, within the testicular open niche could serve as an advantageous model for understanding the temporal and spatial behaviors of aged tissue stem cells within open niches during aging.

### 3.5 Future Perspectives

The age‐related characteristics of SSC clonal dynamics identified in this study are critical, as these may act as a biological filter that selects sperm genomes within the testis, thereby influencing the genetic information passed to the next generation. Accelerated proliferation of SSCs in old testes may lead to the rapid accumulation of mutations, even though the mutation rate in the male germline is generally lower than that in somatic cells (Moore et al. 2021). Additionally, the increased presence of SSC clones with reduced sperm formation in aged testes could decrease the genetic variation among sperm. An important future challenge will be to determine whether non‐sperm‐forming SSC clones have unique genomic characteristics.

Our findings in mice show an age‐related increase in GFRα1^+^ SSC proliferation along with a reduction in spermatid density. Interestingly, healthy elderly men also exhibit an increase in proliferating spermatogonia alongside lower spermatid production efficiency (Pohl et al. 2019), despite differences in SSC systems between the two species—mice maintain continuously proliferating SSCs, whereas a subset of spermatogonia (A‐dark) in humans remains quiescent (Sharma et al. 2019b). Similar trends during physiological aging are observed in other long‐lived mammals, such as bulls, which maintain fertility in old age, yet show a decline in differentiating germ cell density, while proliferatively active undifferentiated spermatogonia persist (Hoffman and Valencak 2020) (Kawahara et al. 2021). Taken together, these findings suggest a comparable, tissue‐level decline in spermatogenic efficiency across mice, humans, and bulls. Therefore, future studies should explore whether the age‐associated changes in clonal fate behavior observed in mouse SSCs are a conserved phenomenon across species. Identifying whether the changes in *Egr4* and *Cops5* expression can serve as reliable markers for evaluating age‐related impacts on SSC function across various species could establish a foundation for developing interventions aimed at preserving reproductive health and maintaining genetic integrity in aging populations.

## 4 Conclusion

In summary, GFRα1^+^ cells in aged testes continue to function as SSCs, showing state heterogeneity, accelerated proliferation, and motility. The SSCs that did not form spermatids exhibited reduced Egr4 and Cops5 expression. In old mice, non–sperm‐forming SSC clones increased and expanded along the seminiferous tubules, marking these clonal changes as hallmarks of stem cell aging in the testicular open niche.

## 5 Materials and Methods

### 5.1 Animals

*GFRα1CreER* ^*T2*^ (Hara et al. 2014), *CAG‐CAT‐EGFP* (Kawamoto et al. 2000), and *GFRα1‐EGFP* (Uesaka et al. 2007) alleles were as previously described. All mice used in this study were heterozygous for two of these alleles and were indicated by their allelic names, with a C57BL/6J background from Japan SLC Inc. (Shizuoka, Japan). Wild‐type C57BL/6J mice were sourced from Japan SLC Inc. and Charles River Laboratories Japan Inc. (Kanagawa, Japan). All animal experiments were approved by the Institutional Animal Care and Use Committee of Tohoku University and the National Institutes of Natural Sciences.

### 5.2 Testicular Cell Counts

Mouse testes were fixed with 4% paraformaldehyde (for immunohistochemistry) or Bouin's solution (for periodic acid Schiff [PAS] staining), dehydrated, mounted in paraffin, cut into 4‐μm sections, and stained with PAS (15792, Muto Pure Chemicals, Tokyo, Japan) and hematoxylin solution (HX90507449, Merck, Darmstadt, Germany), or diaminobenzidine (DAB) (see later). SCO tubules and each type of testicular cell were counted manually under an upright microscope (BX50 and BX61; Olympus, Tokyo, Japan) equipped with a CCD camera (DP71; Olympus). The number of SCO tubules and total seminiferous tubule cross sections quantified was as follows: (SCO tubule cross sections/total tubule cross sections): young, (12/800); middle, (15/1000); and old, (5/600). The Sertoli cells were identified based on their nucleolar morphology (Russell et al. 1990). Spermatogonia, spermatocytes, and spermatids were identified based on their nuclear morphology and PAS localization in the acrosomes of the spermatids. As the number of Sertoli cells per cross section of seminiferous tubule did not differ significantly among the three age groups (Figure S1B,C), we used it to be an index for normalizing germ cell density. Therefore, the density of each spermatogenic cell type was analyzed for three to four testis sections per individual and calculated as the number of cells per number of Sertoli cells.

### 5.3 Section Immunohistochemistry (DAB)

For the visualization of GFRα1^+^, Rarg^+^, and c‐Kit^+^ spermatogonia, the deparaffinized sections were incubated with anti‐GFRα1 (1∶800 dilution; GT15004, Neuromics, Edina, MN, USA), anti‐Rarg (1:200 dilution; 8965, Cell Signaling Technology, Danvers, MA, USA), and anti‐c‐Kit (1:200 dilution; 3074, Cell Signaling) antibodies at 4°C for 12 h. The reaction was visualized using a biotin‐conjugated secondary antibody and an Elite ABC kit (PK‐6100; Vector Laboratories, Newark, CA, USA). The density of each spermatogonial type was analyzed for three to four testis sections per individual and calculated as the number of cells per number of Sertoli cells.

### 5.4 Section Immunohistochemistry (Fluorescence)

The testes were fixed in 4% paraformaldehyde (PFA). Subsequently, they were soaked in 10% and 20% (w/v) solutions of sucrose in phosphate‐buffered saline (PBS) for 2 and 6 h, respectively, at 4°C. They were then cryo‐embedded in OCT compound (4583, Sakura Finetek Japan, Tokyo, Japan), cut into 10‐μm sections using a CM1520 cryostat (Leica, Wetzlar, Germany), dried for 30 min, and washed in PBS at 4°C for 20 min. After blocking, the sections were incubated with anti‐GFRα1 (1∶1000 dilution), anti‐GFP (1:300 dilution; A11122, Invitrogen, Waltham, MA, USA), anti‐GFP (1:1000 dilution; ab13970, Abcam, Cambridge, UK), anti‐Ki67 (1:1000 dilution; ab15580, Abcam), anti‐COPS5 (1:50 dilution; 6895, Cell Signaling Technology), anti‐EGR4 (1:50 dilution; ab198197, Abcam), and anti‐PLZF (1:1000 dilution; AF2944, R&D Systems, Minneapolis, MN, USA) antibodies at 4°C for 12 h. The reaction was visualized with donkey anti‐rabbit IgG Alexa Fluor 488 (1:400 dilution; A21206, Invitrogen), donkey anti‐goat IgG Alexa Fluor 594 (1:400 dilution; A11058, Invitrogen), donkey anti‐chicken IgG Alexa Fluor 488 (1:400 dilution; A78948, Invitrogen), donkey anti‐rabbit IgG Alexa Fluor 594 (1:400 dilution; A21207, Invitrogen), donkey anti‐goat IgG Alexa Fluor Plus 405 (1:400 dilution; A48259, Invitrogen), and Hoechst 33342 (1:5000; 19,172–51, Nacalai Tesque, Kyoto, Japan). Observations were made, and photographs were taken using a BX51 upright fluorescence microscope equipped with a DP74 CCD camera or an FV3000 confocal laser scanning microscope (Olympus).

### 5.5 Whole‐Mount Immunohistochemistry

For whole‐mount immunohistochemistry, immunostaining of whole‐mount seminiferous tubules was performed, as previously described (Nakagawa et al. 2010), using anti‐GFRα1 (1:1000 dilution), anti‐Rarg (1:100 dilution), anti‐GFP (1:300 dilution; Invitrogen), anti‐GFP (1:1000 dilution; Abcam), donkey anti‐rabbit IgG Alexa Fluor 488 (1:400 dilution), donkey anti‐goat IgG Alexa Fluor 594 (1:400 dilution), donkey anti‐chicken IgG Alexa Fluor 488 (1:400 dilution), donkey anti‐rabbit IgG Alexa Fluor 594 (1:400 dilution), and Hoechst 33342 (1:5000 dilution). Observations were made, and photographs were taken using a BX51 upright fluorescence microscope equipped with a DP72 CCD camera. Spermatogonia were judged as belonging to a syncytium when the cell–cell connection was visually detected, based on continuous GFRα1 or GFP staining using a 60× water immersion objective lens. To measure the lengths and types (Type 1 or Type 2) of GFP^+^ patches, an M205FA stereomicroscope with a DFC7000T CCD camera (Leica) was used, and the lengths were measured using ImageJ software (National Institutes of Health, Bethesda, MD, USA). To distinguish between Type 1 and Type 2 patches, we first classified GFP^+^ patches based on the presence (Type 1) or absence (Type 2) of GFP^+^ cells within the luminal space based on whole‐mount observations. We then confirmed the reproducibility of GFP^+^ cell distribution in Type 1 and Type 2 patches by cryosectioning whole‐mount patches. In Type 2 patches, we occasionally observed a small number of GFP^+^ particles without a Hoechst signal within the luminal space, which likely represented degenerating cells (Figure 5H). We also utilized whole‐testis cryosections 3 and 4 months after labeling GFRα1^+^ cells, without isolating the tubules, in the experiments shown in Figure 6 and Figure S4. We prepared serial sections and classified the patches on the cryosections as Type 1 or Type 2, using the same criteria applied to the isolated whole‐mount tubules.

### 5.6 Pulse‐Labeling of GFRα1 ^+^ Spermatogonia

Two‐month‐old (young age group) and 23–28‐month‐old (old age group) *GFRα1‐CreER* ^*T2*^ and *CAG‐CAT‐EGFP* mice were injected intraperitoneally with 0.25 mg/individual 4OH‐tamoxifen (H7904, Sigma‐Aldrich, St. Louis, MO, USA) dissolved in ethanol, dimethyl sulfoxide, and then in sesame oil (25620‐65, Nacalai Tesque). For clonal fate analysis, seminiferous tubules were isolated from testes and subjected to whole‐mount immunofluorescence. To evaluate the differential localization of Egr4 and Cops5 in A_undiff_ within Type 1 and Type 2 patches, testes were processed for fluorescent section immunohistochemistry without isolating the seminiferous tubules.

### 5.7 Intravital Live Imaging

Live imaging of the testes of 25–28‐month‐old GFRα1‐EGFP mice was performed under anesthesia for up to 8 h, as described before, using an epifluorescence IX61WI microscope (Olympus) (Hara et al. 2014; Yoshida et al. 2007). Time‐lapse images were captured at a rate of one frame per 30 min using an Andor Zyla 4.2 Plus sCMOS camera controlled by MetaMorph software (Molecular Devices, San Jose, CA, USA). The movies were also constructed using MetaMorph software. Due to the limited observation time available for old mice, it was not feasible to distinguish whether two or more closely located cells represented isolated cells or syncytia. Therefore, in the first frame of observation, we focused on A_s_ cells that were not surrounded by other cells in the same photograph. The frequencies of cell division and death were measured using live imaging data.

### 5.8 Flow Cytometry and Cell Sorting

CDH1^+^/CD9^+^/c‐Kit^−^ spermatogonia that were isolated via FACS single‐cell suspensions from mouse testes of 3, 19–20, 25–26 months of age were subjected to scRNA‐seq, as described below. The testes were reacted with 5 mg/mL Type 1 collagenase (C5138, Worthington Biochemical, Lakewood, NJ, USA) at 37°C for 12 min, mixed via inversion, and centrifuged twice at 200 × *g* for 5 min. Next, after allowing the samples to react for 2 min with trypsin adjusted to 37°C, FACS buffer (2% FCS/HBSS) was added and pipetted. After separation with a 100‐μm nylon mesh, resuspension in HBSS and centrifugation at 200 × *g* for 5 min were repeated twice. The single‐cell suspension was then incubated with the following antibodies in 0.17 mg/mL DNase I (D5025, Sigma‐Aldrich) on ice for 30 min: Kit‐BV421 (1:250 dilution, 105828, BioLegend), CD9‐PE (1:5000 dilution, 124806, BioLegend), and E‐cad‐PECy7 (1:600 dilution, 47310, BioLegend). The mixture was diluted with HBSS and centrifuged at 200 × *g* for 5 min. Finally, 2000 μL 7AAD‐FACS buffer (1:200 dilution, 420404, BioLegend) was added, and FACS was thereafter performed.

Initially, the cells were analyzed after removing small and large debris in the FSC‐A versus SSC‐A gating, doublets in the FSC‐A versus FSC‐H gating, and 7AAD‐high dead cells. The CDH1^+^/CD9^+^/c‐KIT^−^ cell population was collected in gates based on reporter expression and/or antibody staining (Figure S2A–C).

### 5.9 scRNA‐Seq and Data Analysis

After FACS, centrifugation was performed at 200 × *g* for 5 min. FACS buffer was added, and the mixture was centrifuged at 200 × *g* for 5 min. CDH1^+^/CD9^+^/c‐KIT^−^ mouse spermatogonia were loaded into chromium microfluidic chips with 3'v3 chemistry using a chromium controller (10x Genomics, Pleasanton, CA, USA). All steps used to generate single‐cell libraries were performed according to the manufacturer's recommendations (User Guide CG000204 Rev. D) and libraries were sequenced using a 2 × 150 paired‐end sequencing protocol on an Illumina HiSeq X instrument (Genewiz, South Plainfield, NJ, USA). Trimmed FASTQ files were generated using CellRanger mkfastq (10x Genomics).

The following analyses were performed using the R package Seurat (v4.0.0) (Stuart et al. 2019). For quality control, only cells with 500, < number of genes < 6500; number of unique molecular identifiers (UMIs) < 25,000; and percentage of mitochondrial transcripts < 15% were considered for further analysis. Genes expressed in fewer than three cells, as well as cells expressed in fewer than 200 genes, were excluded. The UMIs were normalized using the NormalizeData function in the R package Seurat.

We selected and used the 6500 most variable genes for linear dimension reduction by applying the RunPCA function. The top 16 PCs were selected and used for the nonlinear dimension reduction implemented by the RunUMAP function. Using this process, 15 clusters were identified. Each cluster was annotated using marker genes related to *Ddx4* expression and differentiation stage (Nakagawa et al. 2021; Suzuki et al. 2021). Additionally, a uniform manifold approximation and projection (UMAP) plot, created based on the data of all individuals, was divided into each age group. Differential gene expression analysis was performed using TCC (Edger) with/without cell cycle genes using an FDR *q*‐value cutoff < 0.01 (Sun et al. 2013; Whitfield et al. 2002).

### 5.10 Quantification and Statistical Analysis

As described in the figure legends, Figures 1B–F,G,I, 4D, 6E,F,H,I and Figure S1A–C,F are presented as mean ± SD or SEM, and the sample size is also displayed in each figure legend. Significant differences among the experimental groups were assessed using a two‐tailed Student's *t*‐test, Tukey's multiple comparison test, or Dunn's multiple comparison test. Violin and dot plots were generated using the R package, Excel, and GraphPad Prism software. Differences were considered statistically significant at *p* < 0.05.

## Author Contributions

K.H. organized the study. T.N., S.Y., and K.H. established key experimental procedures for live imaging and lineage tracing. T.K. and K.H. performed the lineage tracing and live imaging. T.K., Y.K., M.K., and K.T. conducted histological observations and quantified testicular cell density. T.K., M.H., and A.S. carried out genotyping and maintained the aged mice. T.K., S.S., T.N., M.F., and S.Y. performed cell sorting and scRNA‐seq data analyses. T.K. and K.H. prepared the original draft of the manuscript. T.K., S.S., S.Y., and K.H. edited the draft with input from all other authors.

## Conflicts of Interest

The authors declare no conflicts of interest.

## Supporting information


Figures S1–S5.

Data S1.

## Data Availability

Further information and requests for resources, reagents, and biological materials should be directed to and fulfilled by the corresponding author, K.H. (kenshiro.hara.b6@tohoku.ac.jp). scRNA‐seq data have been deposited in the DDBJ and are publicly available as of the date of publication. The accession numbers were as follows: BioProject: PRJDB18908 (PSUB024063) and Run: DRR608871–DRR608888 (tooka_tk‐0003_Run_0001–0035).
