# Supplementary material for: Age‐Dependent Clonal Expansion of Non–Sperm‐Forming Spermatogonial Stem Cells in Mouse Testes
Source: Aging Cell. 2025 Feb 22;24(6):e70019. doi: 10.1111/acel.70019 (PMC12151898; doi:10.1111/acel.70019)
Supplement: Supplementary file 1 — Figures S1–S5. [file ACEL-24-e70019-s002.docx]

**
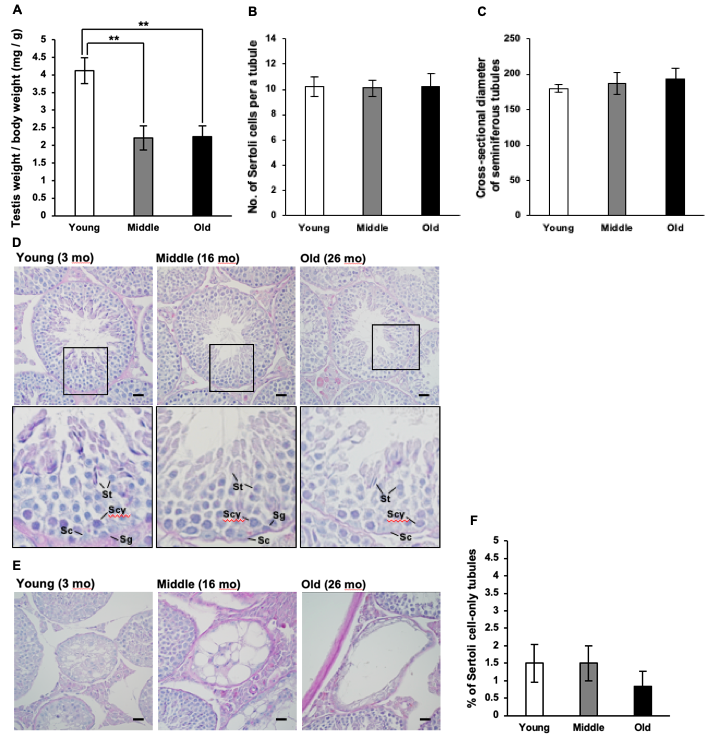
Supplementary figures**

**Figure S1. Age-related changes in the number of testicular cells.**

**(A)** Age-related changes in testis weight / body weight (mg / g). Young, 3–4 mo (16 testes from 8 individuals); Middle, 18–20 mo (18 testes from 9 individuals); Old, 25–28 mo (14 testes from 7 individuals). Values represent averages ± SD of individual data. *p < 0.05, **p < 0.01 (Tukey’s multiple comparison test). **(B)** Age-related changes in Sertoli cell density per seminiferous tubule cross-section. **(C)** Age-related changes in the cross-sectional diameter of the seminiferous tubules. The diameter of one cross-section was defined as the geometric mean of the major and minor axes. **(D)** Upper panels: PAS-H-stained images of seminiferous tubule cross-sections in young (3 months [mo]), middle-aged (16 months), and old (26 months) mice. Lower panels: Enlarged views of the boxed areas indicated in the upper panels. Cells were classified as those with a representative morphology of the indicated cells. Abbreviations: Sc, Sertoli cells; Sg; spermatogonium; Scy; spermatocyte; St; spermatid. **(E)** PAS-H-stained images of cross-sections of Sertoli cell-only (SCO) tubules in young (3 months), middle-aged (16 months), and old (26 months) mice. **(F)** The Percentage of SCO tubules among all seminiferous tubules in each section. **(B, C, F)** Young, 3 mo (n = 4); Middle, 15–18 mo (n = 5); Old, 26 mo (n = 3). Averages ± SD are shown. Tukey’s multiple-comparison test was performed; however, no significant differences were observed. **(D, E)** Scale bar: 20 μm.


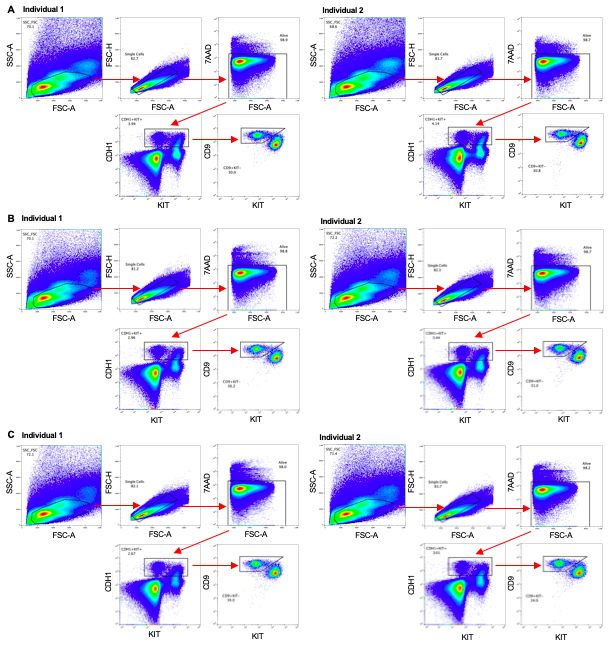
**Figure S2. FACS procedure and results.**

**(A–C)** FACS procedure and single-cell RNA sequencing results for **(A)** young, **(B)** middle-aged, and (**C**) old mice. Detailed cellular methods corresponding to these data are described in Materials and Methods.

**
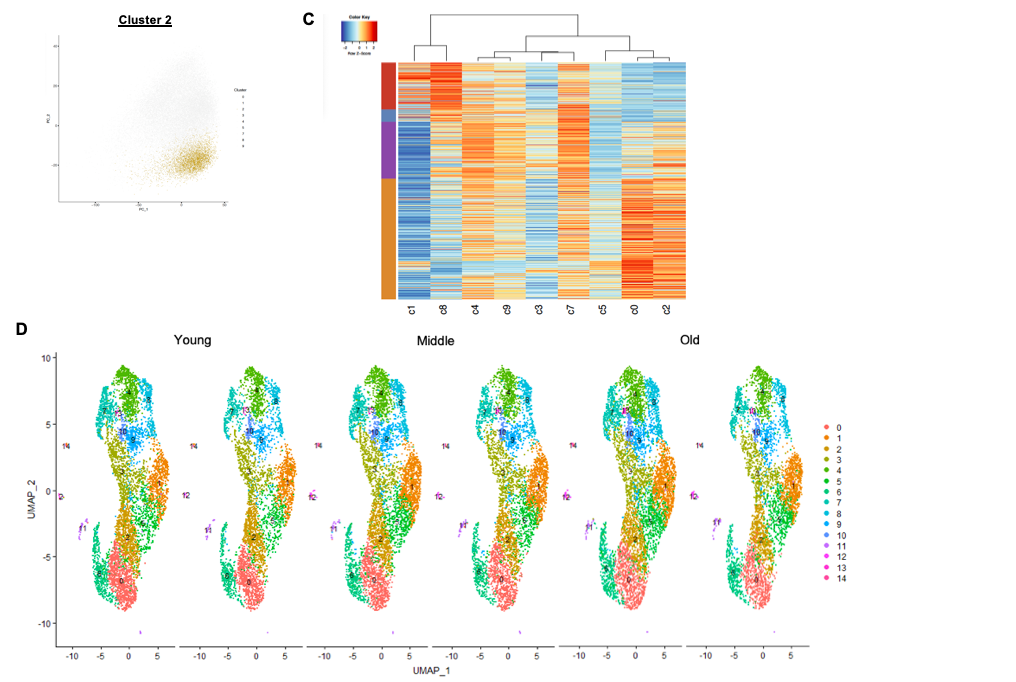

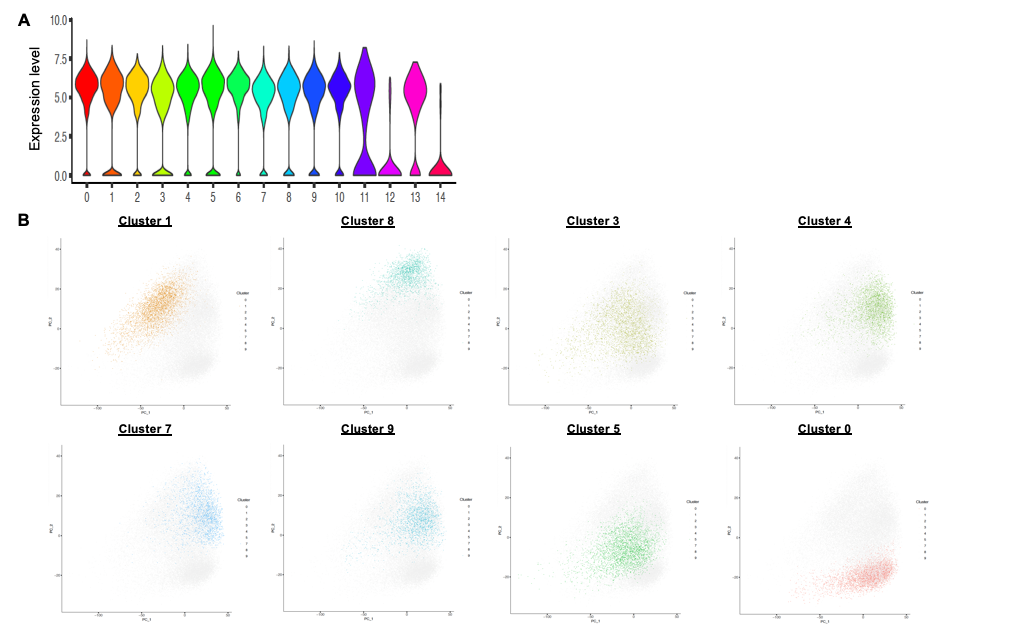
Figure S3. Age-related changes in heterogeneity of gene expression in undifferentiated spermatogonia populations.**

**(A)** Violin plot showing *Ddx4* expression in each cluster obtained from the single-cell RNA sequencing data. Clusters other than 11, 12, and 14, which had a high proportion of cells with expression levels between 0 and 2.5, were used in subsequent analyses. **(B)** Principal component and **(C)** hierarchical clustering analyses using genes^1^ highly expressed in Plvap^+^ (201 genes), Plvap^+^/Sox3^+^ (56 genes), Sox3^+^ (246 genes), and Ngn3^+^ (525 genes) clusters. Results of these two analyses show that c5 is closer to cluster c0c2 than it is to cluster c3c4c7c9. **(D)** Uniform manifold approximation and projection (UMAP) plot for each individual.


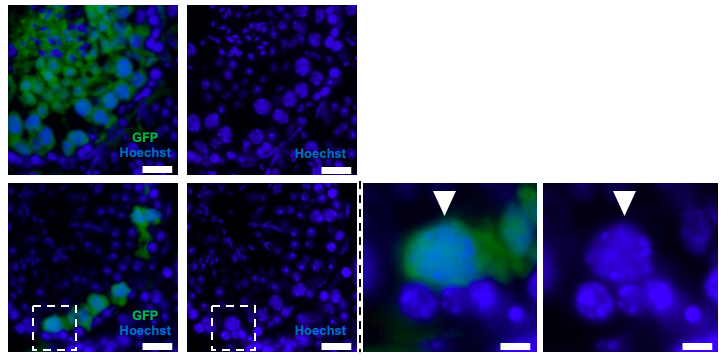
**Figure S4. Long-term dynamics of GFRα1+ cell-derived clones in aged mice.**

Cross-sectional GFP (green) and Hoechst (blue) staining of type 1 (upper) and type 2 (lower) clones at four months postinduction in old mice. Dotted rectangles indicate an enlarged area shown in right panels. (Lower, Right) Enlargement of dotted rectangles in left panels showing GFP+ pachytene spermatocytes in type 2 clones. Arrowhead indicates pachytene spermatocyte. Scale bars: (left) 20 μm; (right) 5 μm.


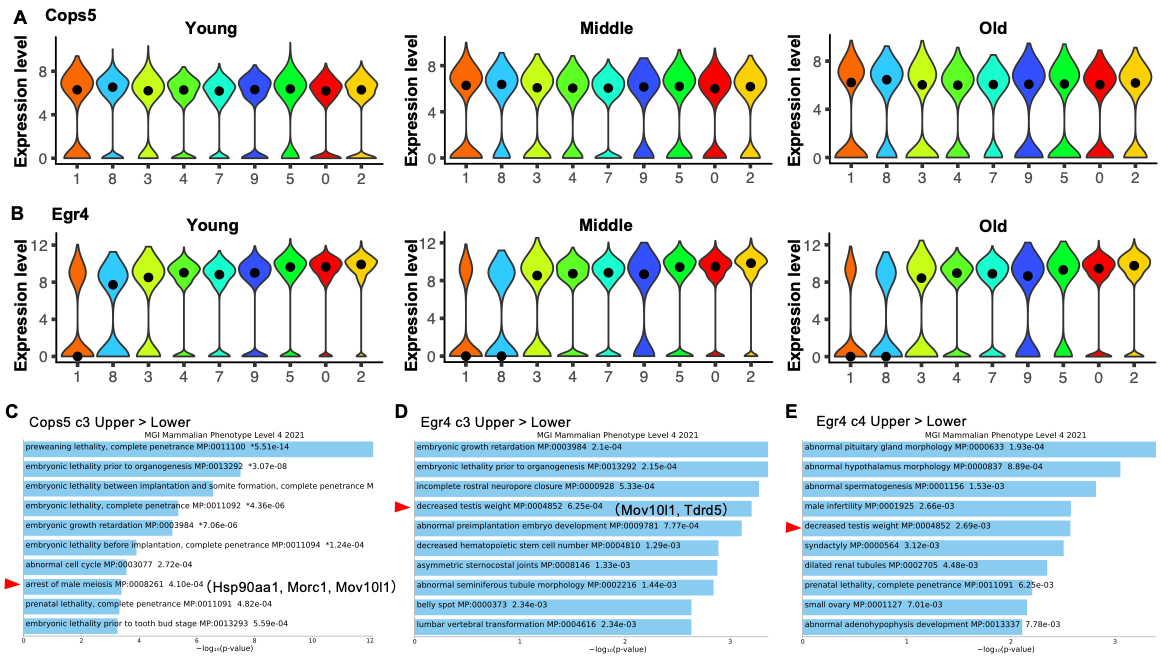
**Figure S5. Screening for factors associated with the age-related increase in type 2 spermatogonia.**

**(A–B)** Violin plots showing the expression of **(A)** *Cops5* and **(B)** *Egr4* in undifferentiated spermatogonia clusters from young, middle-aged, and older mice. Numbers on the horizontal axis indicate cluster numbers and are arranged from left to right in order of the differentiation state. Black dots indicate the median values in each cluster. This indicates that the proportion of cells with expression levels between 0 and 4 increased with age.

**References**

1. Nakagawa, T., Jörg, D.J., Watanabe, H., Mizuno, S., Han, S., Ikeda, T., Omatsu, Y., Nishimura, K., Fujita, M., Takahashi, S., et al. (2021). A multistate stem cell dynamics maintains homeostasis in mouse spermatogenesis. *Cell Rep* *37*, 109875. 10.1016/j.celrep.2021.109875.
