# Supplementary material for: Age‐Dependent Clonal Expansion of Non–Sperm‐Forming Spermatogonial Stem Cells in Mouse Testes
Source: Aging Cell. 2025 Feb 22;24(6):e70019. doi: 10.1111/acel.70019 (PMC12151898; doi:10.1111/acel.70019)
Supplement: Supplementary file 2 — Data S1. [file ACEL-24-e70019-s001.zip › TableS1,S2, VideoS1, S2 caption 250207.docx]

**Table S1. Raw data from clonal fate analysis of GFRα1^+^ cells (related to Fig. 3)**

Table of the number of GFRα1^+^ and GFRα1^-^ units in each clonal population. The spreadsheet was divided by the time points analyzed. Clone numbers are shown without distinguishing individuals. No units longer than Aal9 were observed in GFRα1^+^ units.

**Table S2. List of DEGs obtained by DEG analysis (related to Fig. 6A)**

Table for the list of DEGs between Young and Old in Cluster 5 and their expression levels in each age group. Genes with more than 2-fold difference in expression levels and p-values less than 0.05 between Young and Old are shown in alphabetical order. Expression levels for each age group are shown log_2_-fold. Cops5 and Egr4 (red) showed a significant difference in the proportion of cells expressed in Type 2 A_undiff_ compared to Type 1 (Fig. 6D-I).

**Video1. Cell division of GFRα1-GFP+ cells in old age (related to Fig. 4B)**

An example of cell division observed by live imaging analysis of GFRα1-GFP+ cells in old mouse testis. This movie was created using images taken every 30 minutes for 4.5 hours.

**Video2. Migration of GFRα1-GFP+ cells in old age (related to Fig. 4E)**

An example of cell migration observed by live imaging analysis of GFRα1-GFP+ cells in old mouse testis. This movie was created using images taken every 30 minutes for 8 hours.
